# Hg(II) immobilization and detection using gel formation with tetra-(4-pyridylphenyl)ethylene and an aggregation-induced luminescence effect

**DOI:** 10.1038/s41598-023-29431-0

**Published:** 2023-02-06

**Authors:** Bing Hu, Taibao Wei, Yanjun Cui, Xia Xu, Qiao Li

**Affiliations:** 1 College of Science, Gansu Agricultural University, Lanzhou, 730070 Gansu People’s Republic of China; 2 College of Chemistry and Chemical Engineering, Northwest Normal University, Lanzhou, 730070 Gansu People’s Republic of China; 3 College of Chemical Engineering, Lanzhou University of Arts and Science, Lanzhou, 730000 Gansu People’s Republic of China

**Keywords:** Chemistry, Materials science

## Abstract

Tetra-(4-pyridylphenyl)ethylene (TPPE), featuring an aggregation-induced luminescence effect (AIE), has been synthesized and used for selective detection of Hg^2+^ in DMF/H_2_O (3:7, v/v) binary solutions. There was a color change from colorless to yellow in the detection of the Hg^2+^ ions, in addition to an increased fluorescence emission. This shows that TPPE will function as an excellent “turn-on” fluorescence probe in the detection Hg^2+^. Moreover, the interference of Al^3+^, Ba^2+^, Mn^2+^, Ca^2+^, Fe^3+^, Cu^2+^, Ag^+^, Cd^2+^, Co^2+^, Ni^2+^, Mg^2+^, Pb^2+^, Zn^2+^, and Cr^3+^ ions was found to be negligible under optimized solvent conditions. Cysteine and EDTA were also found to form TPPE-based fluorescent switches with the Hg^2+^ ions. The practical use of the TPPE sensor was also demonstrated by using a specific test kit. Characterization using FT-IR, NMR titration, UV titration, EDS, and HR-MS techniques showed that Hg^2+^ will form a 1:1 complex with TPPE. Also, the observation of a Tyndall effect, in addition to UV absorption and fluorescence spectra, did clearly demonstrate the presence of an AIE. More noteworthy, TPPE and Hg^2+^ were found to form a metal–organic gel (MOG) in the DMF solution. The SEM, TEM, ICP, and Zeta potential analyses confirmed that the fluorescent MOG could further adsorb an excess of Hg^2+^ ions. The BET analyses revealed that the MOG showed a type IV-H3 hysteresis loop according to the International Union of Pure and Applied Chemistry classification. The results of the XRD analysis and of the spectroscopic titrations show that a π–π stacking may be the auxiliary driving force for the gel formation, after that a coordination has taken place. These results indicate that further research on structurally simple metal ion fluorescent probes, which are based on the AIE, is promising for the achievement of a simultaneous fluorescent detection and adsorption of heavy metal pollutants.

## Introduction

In the past decades, since Tang et al. proposed the concepts of “Aggregation-Induced Emission” (AIE), the applications on AIE have gained increasing attentions^1^. The AIE is a phenomenon that some compounds showed weak/no fluorescence emission in good solvent but highly emissive in the aggregated state (e.g. in aqueous media)^2^. In the AIE state, the intramolecular rotations are restricted through variety of molecular interactions including hydrogen bonding, π–π interactions and steric effect, resulting in the elimination of nonradiative transitions and promotion of radiative transitions which affords strong emission^3^. Based on this pioneering research and other numerous fundamental studies, AIE has shown widespread applications in bioimaging^4,5^, organic lightemitting diodes (OLED)^6^, and also towards the sensing of cations and anions^7,8^.

Mercury and mercury ions are highly toxic pollutants that are currently found in many environments^9,10^. There are several detection methods to target Hg^2+^, such as atomic absorption spectroscopy (AAS)^11^, inductive couple plasma mass spectrometry (ICP-MS)^12^, electrochemical methods^13^, optical probes^14^, etc. Among these ones, optical probes display interesting advantages such as high sensitivity, simplicity, visualization, and ease of operation^15,16^. In particular, optical probes that are primarily based on the aggregation-induced luminescence effect (AIE) have attracted large attention due to their enhanced luminescence at high concentrations^17,18^.

The coordination-induced emission (CIE) is complementary to aggregation-induced luminescence. Although metal coordination results in a wider separation of the chromophores for molecular complexes, the coordination of M^n+^ ions by the chromophore ligand (in coordination polymers (CPs)) results in shorter separations, as well as in an aggregation-induced effect and an enhanced luminescence^19,20^. In recent years, CP-based Hg^2+^ fluorescent probes have become extensively investigated^21,22^. However, most of these studies are based on the fluorescence quenching (turn-off) action. Compared to turn-off probes, fluorescence enhancing (turn-on) probes are superior from the viewpoints of sensitivity, simplicity of the detection mechanism, and a reduced interference^23,24^.

Research in metallogels is a new field within supramolecular chemistry. The synthesis of metallogels is similar to the synthesis of metal compounds. The introduction of metal components into gelators provides an effective way to develop soft materials with advantageous properties, such as luminescence, adsorption, separation, etc^25–27^.

It is worth noting that the adsorption of highly toxic heavy metals is attracting a growing attention. Moreover, traditional fluorescent sensors are able to detect Hg^2+^ ions. However, they cannot isolate these Hg^2+^ ions. On the other hand, certain new mercury ion detection and adsorption materials have the disadvantages of a complex structure and cumbersome synthesis steps^28^. Therefore, the design and development of a structurally simple and easily prepared material that will not only perform a high-sensitivity and highly-selective fluorescence detection of Hg^2+^, but also immobilize this ion, is highly desirable.

Because of these considerations, and of our group’s longstanding interest in ion-responsive supramolecular compounds^25^, we have in the present study investigated the use of tetra-(4-pyridylphenyl)ethylene (TPPE) (which features an AIE^29^) as a Hg^2+^ detection probe^30^. We have then shown that this system can achieve both a high-selective and a high-sensitive fluorescence detection of Hg^2+^ in DMF/H_2_O (3:7, v/v) binary solutions. Moreover, the 1:1 combination of TPPE-Hg has been found to form a metal–organic gel at high concentrations, by which it is possible to further absorb Hg^2+^ ions and, thus, simultaneously remove Hg^2+^ from the DMF.

**Figure 1:**
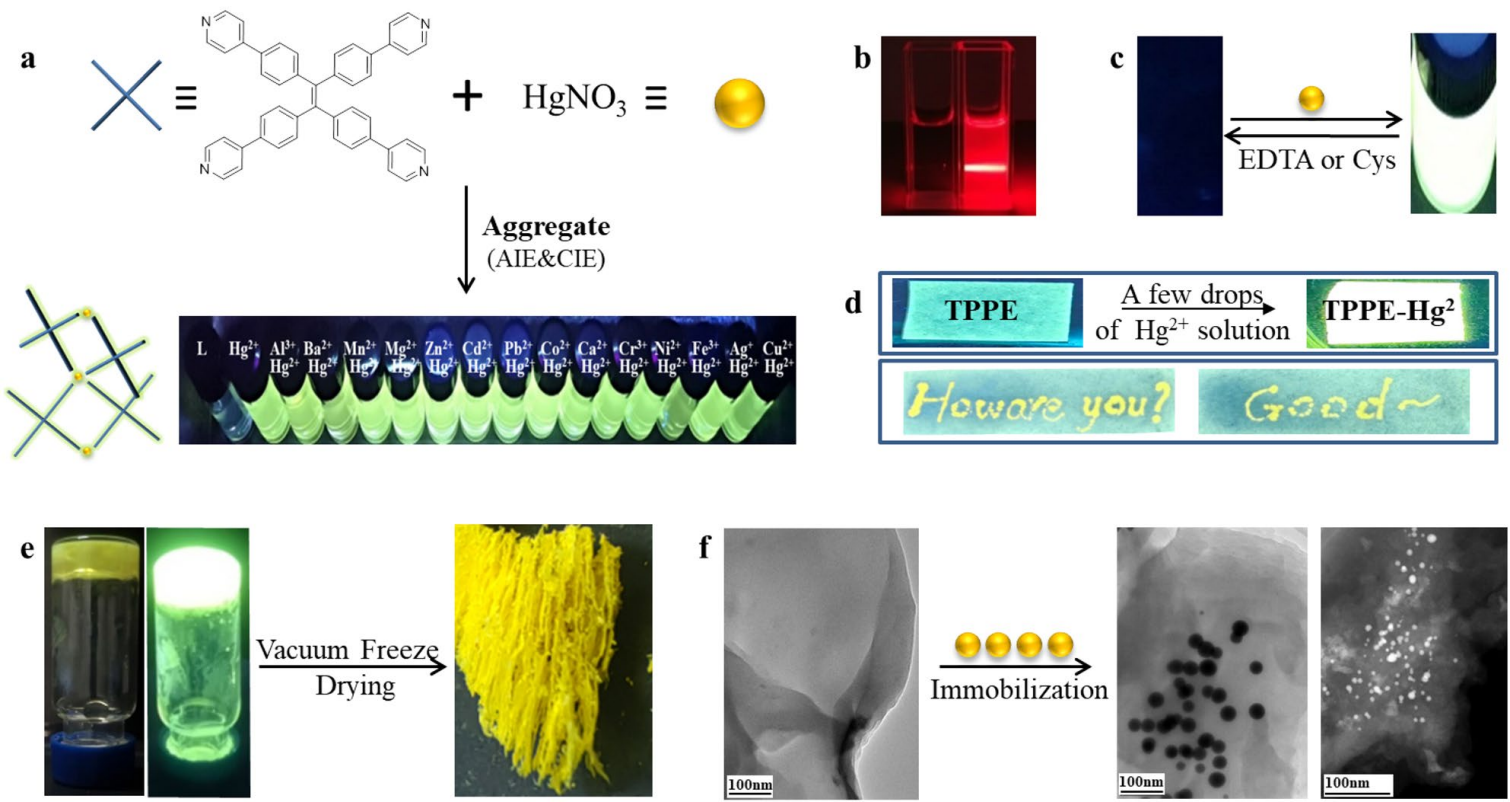
(**a**) Demonstration of the detection of Hg^2+^ ions by the use of TPPE. (TPPE: 2.0 × 10^–5^ M, cations: 4.0 × 10^–4^ M). (**b**) Image of the Tyndall effect before (left) and after (right) the addition of Hg^2+^ to TPPE. (TPPE: 2.0 × 10^–5^ M, Hg^2+^: 4.0 × 10^–4^ M). (**c**) A cyclic fluorescent switc h composed of TPPE-Hg and EDTA/Cys under UV light. (**d**) The effect of TPPE as a Hg^2+^ detection kit. (**e**) At certain concentrations, TPPE and Hg^2+^ can generate luminescent MOG in DMF solution. Xerogels exhibit a loose multi-layered structure. (TPPE: 9.6 × 10^–3^ M, Hg^2+^: 3.8 × 10^–2^ M). (**f**) Bright and dark field TEM image of the xerogel constructed with a TPPE:Hg of 1:4.

Based on the results in the present study, it is possible that the Hg-N coordination interaction preferentially guides the aggregation-induced luminescence phenomenon, and that TPPE forms a 1:1 coordination polymer with Hg^2+^. The metal ions are then tetragonally coordinated by the N atoms in their common tetrahedral environment^31–33^. With an increase in concentration, a π-π stacking will promote the gel construction, and the multi-layer stacked polymer gel network will be able to further adsorb an excess of HgNO_3_ (Fig. 1).

## Results

### Fluorescence and colorimetric identification of Hg^2+^ by using TPPE

As a novel AIE sensor and potential ligand, the interaction of TPPE with various metal ions has been explored by using UV–vis absorption and fluorescence spectroscopies. This resulted in excellent fluorescence and colorimetric recognitions of Hg^2+^ ions.

#### Solvent selection

**Figure 2:**
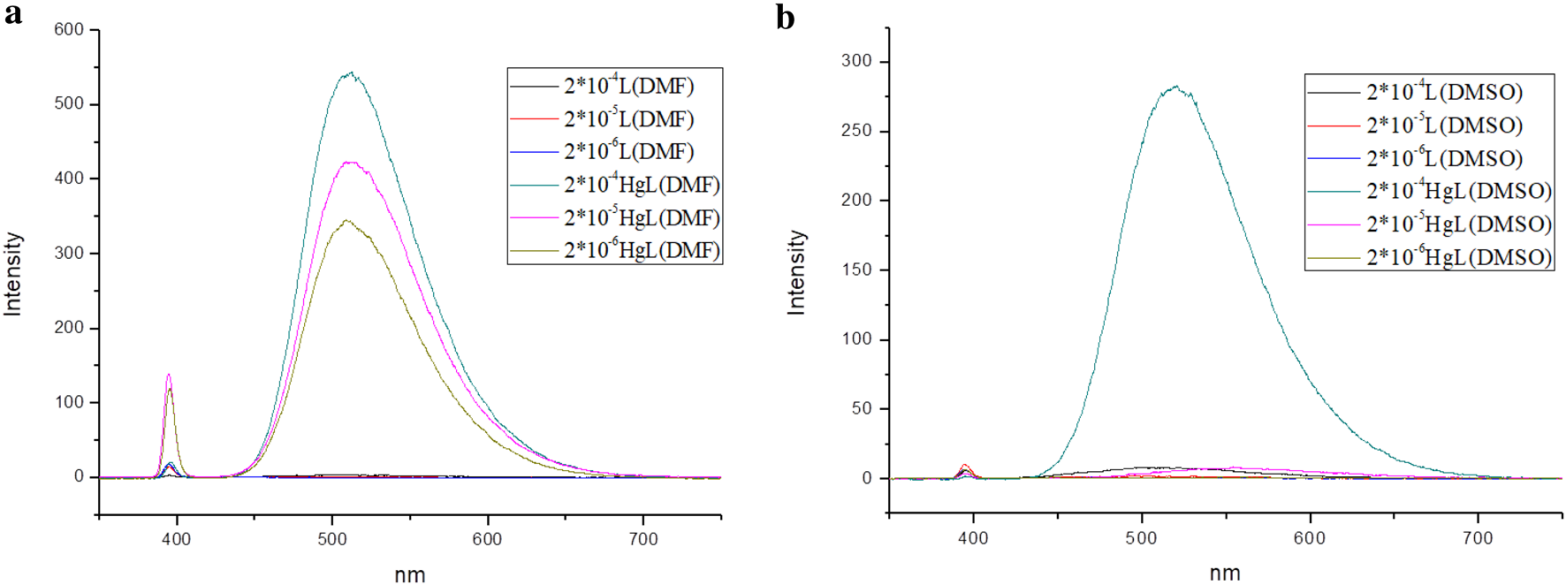
Fluorescence spectra for different concentrations of TPPE in (**a**) DMF and (**b**) DMSO, before and after the addition of Hg^2+^ ions (λ_ex_  = 394 nm).

The TPPE-Hg^2+^ interaction was primarily investigated in DMF and DMSO at the concentrations of 2 × 10^–4^ mol L^−1^, 2 × 10^–5^ mol L^−1^, and 2 × 10^–6^ mol L^−1^. The detection sensitivity was found to be much higher in DMF than in DMSO (Fig. S1, λ_ex_ = 365 nm). The solution fluorescence emission maximum was found to be slightly red-shifted (508 nm in DMF, 516 nm in DMSO; Fig. 2a,b, λ_ex_ = 394 nm) with an increase in solvent polarity. This is an indication of a small solvation effect. Furthermore, the fluorescence of the solution when positioned under a UV lamp was found to be stable with time, with no noticeable change in color. This is an indication of a complex with good photostability.

The corresponding ultraviolet absorption spectrum showed the appearance of a new peak (at 341 nm) after the addition of Hg^2+^ ions to DMF. This is an indication of the formation of a new compound. For DMSO, the shape of the absorption curve was observed to change with a change in concentration after the addition of Hg^2+^. This indicates that the solute has interacted with the solvent and that the system stability is poor (Fig. S2a,b). Therefore, DMF has in the present study been selected as the TPPE solvent in the detection of Hg^2+^ ions.

#### Cation selectivity in DMF

**Figure 3:**
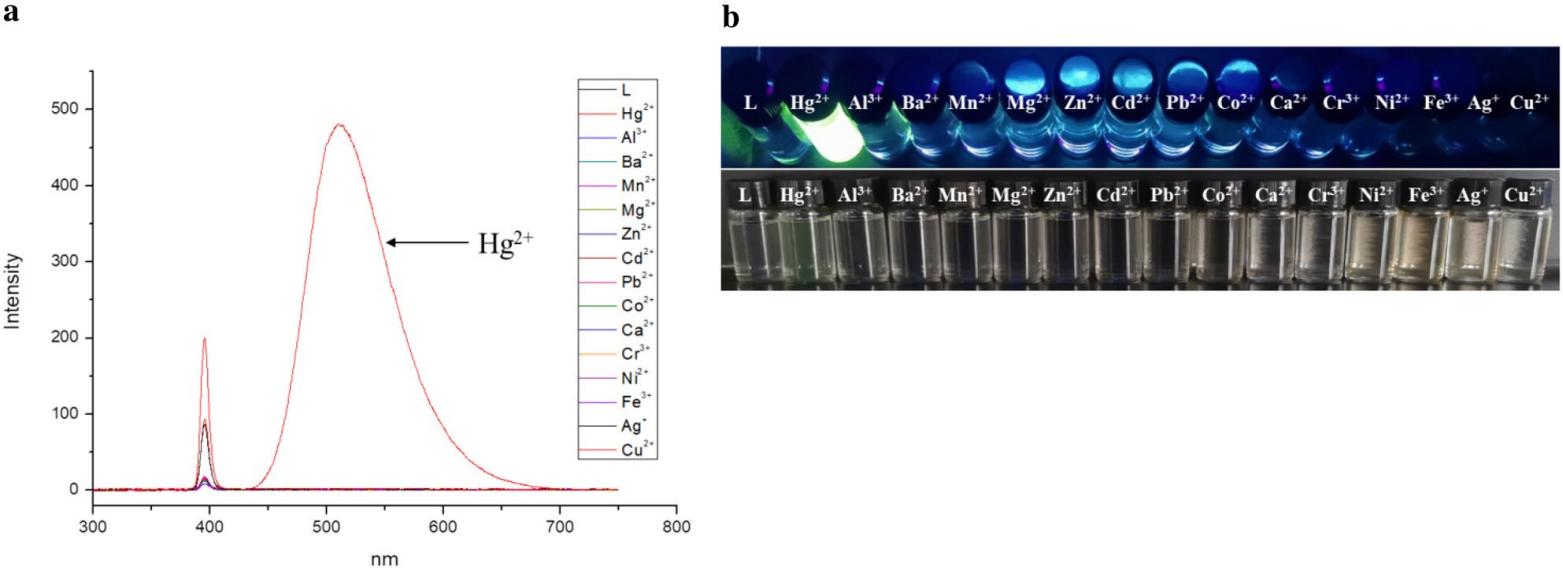
(**a**) Fluorescence spectra for the selective recognition of Hg^2+^ by TPPE in DMF (λ_ex_  = 394 nm). (**b**) Fluorescence and colorimetric identification of Hg^2+^ in DMF by TPPE under UV light (λ_ex_  = 365 nm) and visible irradiation (TPPE: 2.0 × 10^–5^ M, cations: 4.0 × 10^–4^ M).

The TPPE sensing ability for various metal ions (Hg^2+^, Al^3+^, Ba^2+^, Mn^2+^, Ca^2+^, Fe^3+^, Cu^2+^, Ag^+^, Cd^2+^, Co^2+^, Ni^2+^, Mg^2+^, Pb^2+^, Zn^2+^, and Cr^3+^) has in the present study been examined by using fluorescence spectroscopy in the characterization of DMF. Except for Hg^2+^, the addition of any of the above metal ions to the TPPE solution did not lead to any significant change in the fluorescence spectra (as shown in Fig. 3a). Furthermore, Fig. 3b shows an evident color change (to yellow) for the Hg^2+^-containing solution when exposed to visible irradiation, while none of the other ions led to any evident color change in the presence of TPPE.

#### *Hg*^*2*+^*detection in the presence of other cations in DMF and in DMF/H*_*2*_*O binary solutions*

**Figure 4:**
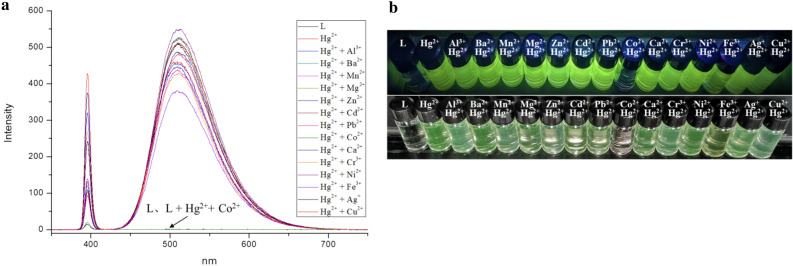
(**a**) Fluorescence spectra for the detection of Hg^2+^ by using TPPE in the presence of other cations in DMF (λ_ex_  = 394 nm). (**b**) Fluorescence and colorimetric identification of Hg^2+^ by using TPPE in the presence of other cations in DMF under UV light (λ_ex_  = 365 nm) and visible irradiation (TPPE: 2.0 × 10^–5^ M, cations: 4.0 × 10^–4^ M).

Further studies of the interference by the above-mentioned cations on the Hg^2+^ detection show, apart from Co^2+^, a minor effect. As can be seen in Fig. 4, the TPPE-Hg^2+^ luminescence is completely quenched for the Co^2+^ ion. This result is consistent with the literature—the Co^2+^ ion is known to establish a strong interaction with TPPE^34,35^.

**Figure 5:**
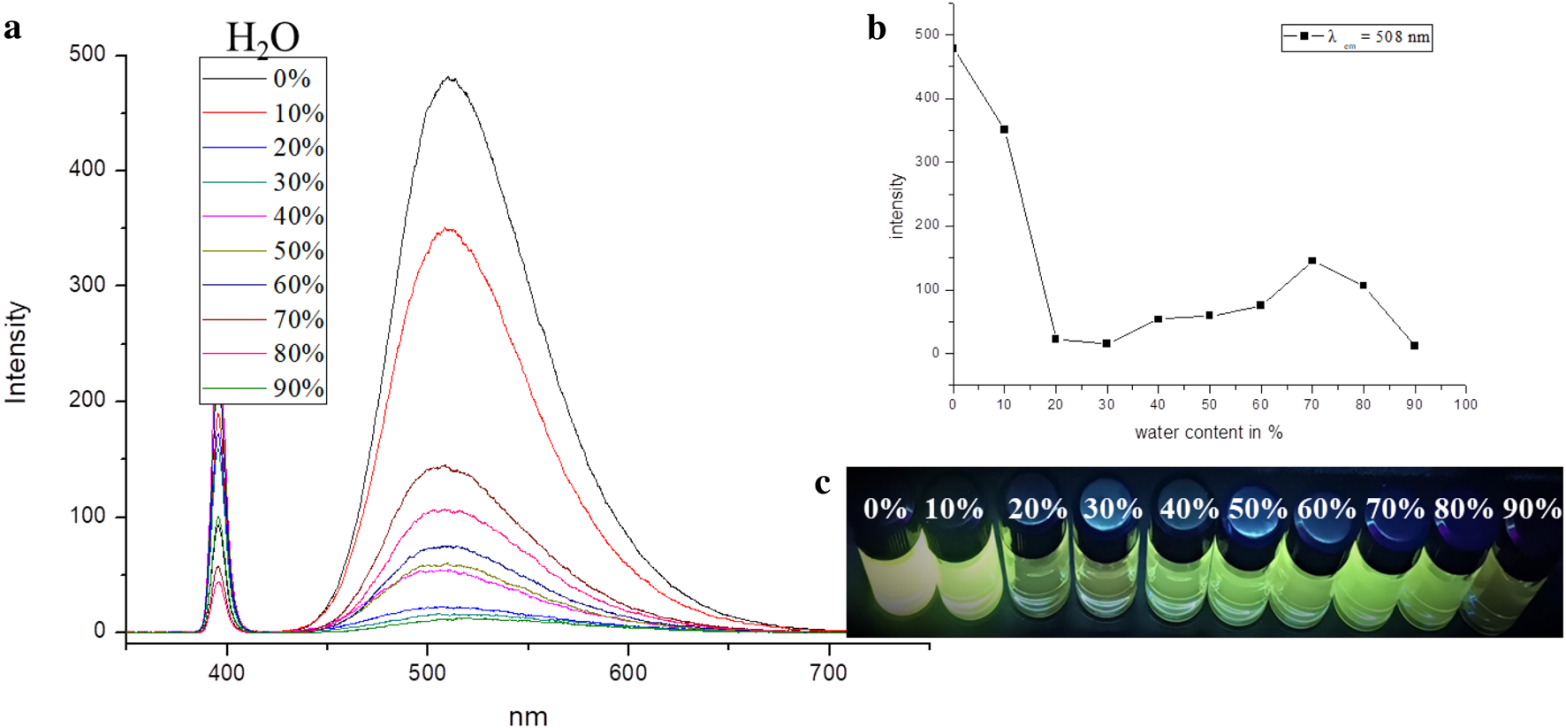
(**a**) Variations in the fluorescence spectrum of Hg^2+^ (in DMF) with different water contents, as detected by using TPPE (λ_ex_  = 394 nm). (**b**) Line plot of emission intensity at 508 nm as a function of water content. (**c**) Image of the fluorescence intensity changing with water content (λ_ex_  = 365 nm).

By considering the fact that the Hg^2+^ pollutant is mainly found in an aqueous environment, and that water, as a very polar solvent, will weaken the metal–ligand interaction and possibly altering the AIE, we have further investigated the Hg^2+^ detection by using TPPE in a DMF/H_2_O binary solution. As shown in Fig. 5, the fluorescence has substantially decreased with an increase in the volume fraction of water (from 0 to 30%). However, it became increased for an increase in the water content from 30 to 70%. Furthermore, the absorption peak did undergo a 7 nm red-shift when going from 0 to 70% water content, which was caused by an aggregation in the less effective water solvent. However, when the water content exceeded 70%, the fluorescence intensity decreased again, which was most probably due to solubility limitations. The same experiment for the DMF/EtOH binary solution showed that the fluorescence emission intensity did not significantly change when altering the volume fraction of EtOH (i.e., ethanol) (Fig. S3). This is due to the improved solubility of TPPE in ethanol.

#### Mercury-selective recognition by adjusting the solvent in the presence of other cations

**Figure 6:**
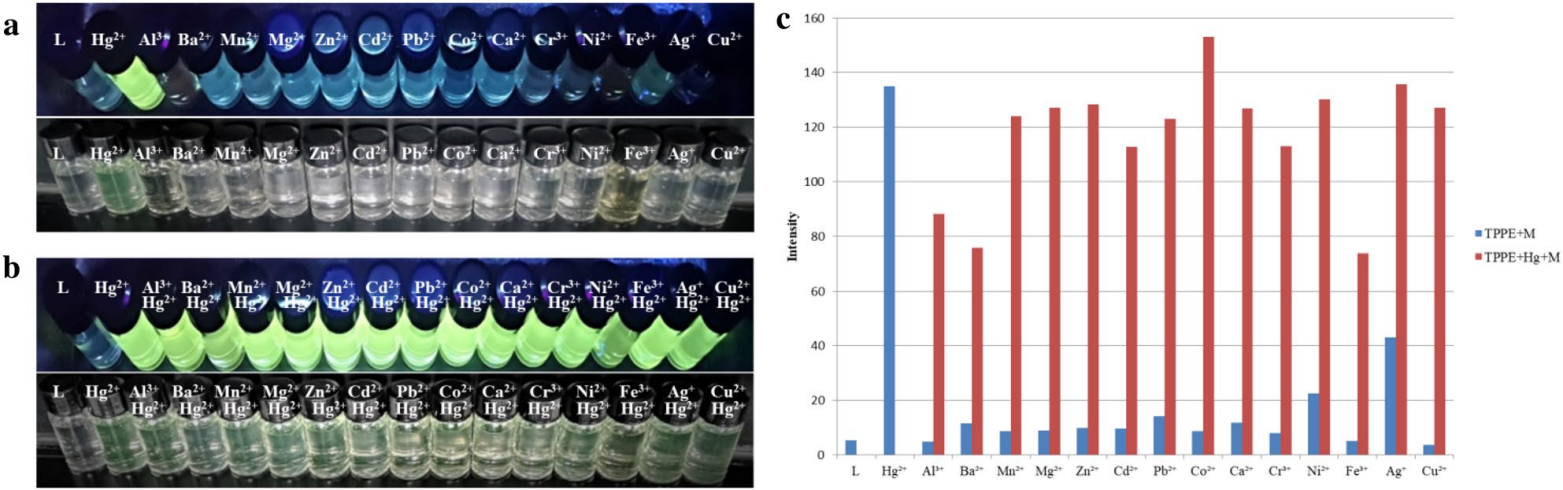
(**a**) Recognition effect by TPPE for various cations in a DMF/H_2_O (3:7, v/v) binary solution, under UV light (λ_ex_  = 365 nm) and visible irradiation. (**b**) Recognition effect by TPPE for Hg^2+^ in the presence of other cations in a DMF/H_2_O (3:7, v/v) binary solution, under UV light (λ_ex_  = 365 nm) and visible irradiation. (**c**) Fluorescence recognition histograms for TPPE and various cations, and for TPPE and Hg^2+^ in the presence of other cations (TPPE: 2.0 × 10^–5^ M, cations: 4.0 × 10^–4^ M).

After determining the optimal solvent ratio for the Hg^2+^ detection in a DMF/H_2_O solution, an identification and interference test for the above-listed 14 metal cations was carried out (Fig. 6). Notably, the good fluorescence and selective recognition of Hg^2+^ were observed for the DMF/H_2_O (3:7, v/v) binary solution, and the presence of the other 14 cations, including Co^2+^, did not significantly interfere with the Hg^2+^ detection.

To further explore the relationship between the water fraction and the Co^2+^ interference, the luminescence of a solution containing both Hg^2+^ (4.0 × 10^–4^ M) and Co^2+^ (4.0 × 10^–4^ M) was measured as a function of the water fraction (Fig. S4). The results demonstrated that Co^2^ showed in principle no interference in the Hg^2+^ detection for water contents larger than 60%. This is consistent with Pearson's HSAB theory^36^. The Co^2+^ ion, being a harder cation, shows a larger affinity towards the harder H_2_O base. Thus, the solvated Co(H_2_O)_6_^2+^ ion shows a much smaller affinity towards TPPE, whereas Hg^2+^, which is a quite soft Lewis acid, shows a very low affinity towards water coordination and, thereby, binds much easier with the softer TPPE donor. In addition to the above reasons, when Hg^2+^ forms a σ-coordination bond with TPPE, the 5d electrons of Hg^2+^ can enter the π*-antibonding orbital of pyridine ring due to its u-symmetry, which matches the u-symmetry π*-antibonding orbital of pyridine ring, thus forming a π-back bonding^37^. While other soft acid cation, such as Ag^+^, have a larger radius (126 nm) than Hg^2+^ (102 nm), so the d-orbitals are distributed over larger region and are less likely to form good π-back bonding. Another soft acid example is Pb^2+^, whose d-orbital is an inner orbital and cannot participate in the formation of the π-back bonding. Thus, Hg^2+^ achieves a single selectivity for TPPE in binary solvents.

#### *Response of TPPE-Hg*^*2*+^*to common amino acids in DMF/H*_*2*_*O (3:7, v/v) binary solutions*

**Figure 7:**
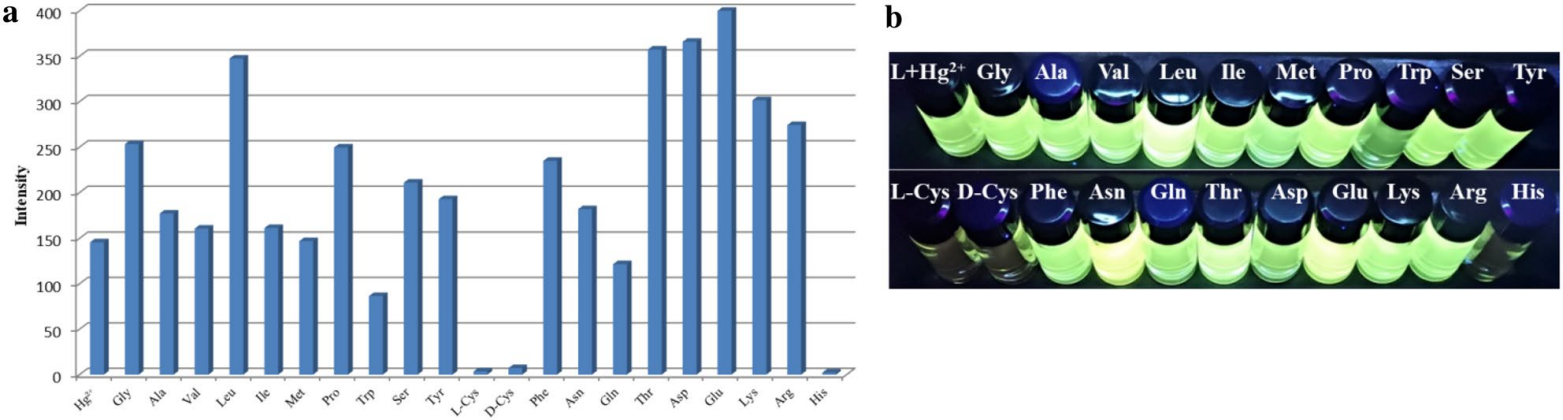
(**a**) Fluorescence response histogram and (**b**) UV images for TPPE-Hg with 21 amino acids (TPPE: 2.0 × 10^–5^ M; Hg^2+^ and amino acids: 4.0 × 10^–4^ M; λ_ex_  = 365 nm).

When considering that certain amino acids are insensitive for the toxicity of heavy metals such as mercury, cadmium, and lead, the fluorescence properties of the TPPE-Hg^2+^ adduct in DMF/H_2_O (3:7, v/v) have been measured in the presence of 21 common amino acids. These experiments showed that the addition of histidine, or cysteine (both L- and D-,) will induce fluorescence quenching, while the other 18 amino acids showed a much smaller effect. The reason is that both the imidazole group of histidine, and the sulfhydryl group of cysteine, can coordinate to the Hg^2+^ ion. This leads to a competition with the coordination TPPE to Hg^2+^ and, thereby, to fluorescence quenching. This indicates that the TPPE-Hg^2+^ complex can be used for the detection of histidine and cysteine (Fig. 7).

#### Fluorescence switch by the action of EDTA or cysteine

**Figure 8:**
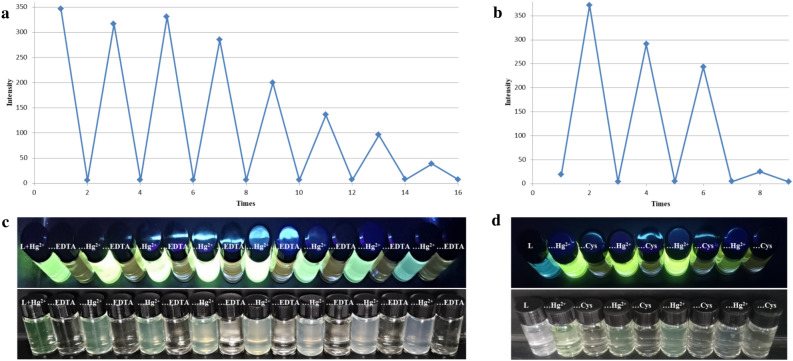
(**a**) Graph of a cyclic fluorescent switc h composed of TPPE-Hg and EDTA. (**b**) Graph of a cyclic fluorescent switch composed of TPPE-Hg and Cys. (**c**) Images of a cyclic fluorescence switch composed of TPPE-Hg and EDTA under UV light (λ_ex_  = 365 nm) and visible irradiation. (**d**) Images of a cyclic fluorescent switch composed of TPPE-Hg and Cys under UV light (λ_ex_  = 365 nm) and visible irradiation.

As a classical metal ion chelating agent, ethylenediaminetetraacetic acid (EDTA) may deprive TPPE from the Hg coordination and cause a fluorescence quenching of TPPE-Hg^2+^. This type of fluorescence quenching has here occurred when an equimolar amount of EDTA was added to the TPPE-Hg^2+^ solution in DMF/H_2_O (3:7, v/v). However, the fluorescence was recovered upon addition of more Hg^2+^ ions. A repetition of alternate EDTA and Hg^2+^ additions led to an occurrence of multiple fluorescence switches (Fig. 8). A similar result was also obtained by using cysteine as a fluorescence quencher instead of EDTA. However, the cysteine-based switching system was less stable. It only allowed three switching cycles.

### Tyndall experiment

**Figure 9:**
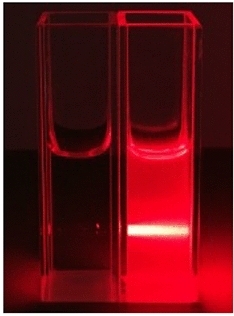
Image of the Tyndall effect before (left) and after (right) the addition of Hg^2+^ to TPPE. (TPPE: 2.0 × 10^–5^ M, Hg^2+^: 4.0 × 10^–4^ M).

The aggregation behavior of the TPPE probe, in the presence of Hg^2+^ ions, was also investigated by performing a Tyndall experiment. TPPE-Hg^2+^ showed a strong Tyndall effect for a TPPE concentration of 2.0 × 10^–5^ M in DMF (Fig. 9). In contrast, there was no apparent Tyndall effect for pure TPPE at a concentration of 2.0 × 10^–5^ M. The Tyndall experiment has established the presence of aggregations in TPPE-Hg^2+^, which leads to the AIE and fluorescence enhancement.

### ^1^H NMR titration and HR-MS

**Figure 10:**
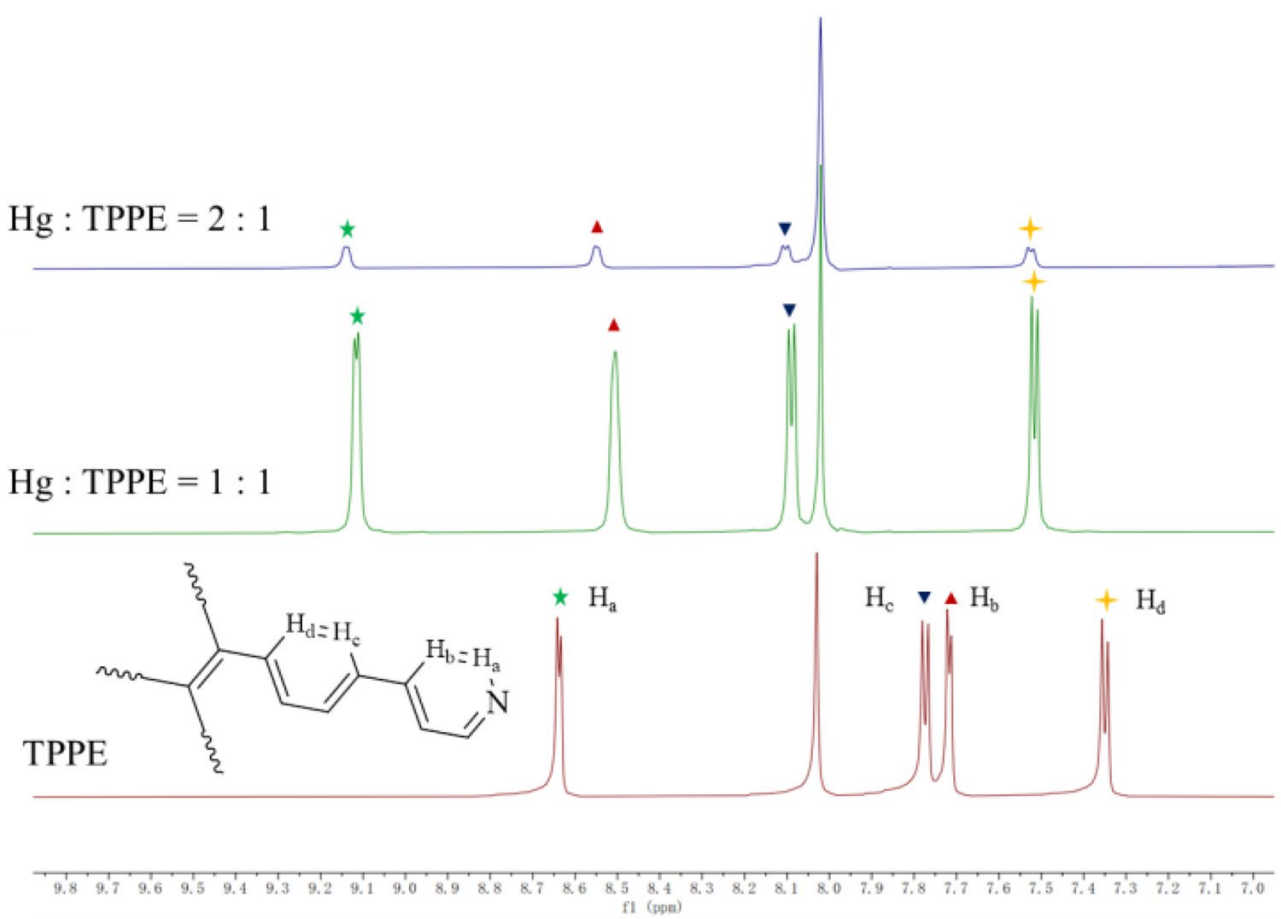
^1^H NMR titration of TPPE by Hg^2+^ with DMF-d_7_ as a solvent.

To further investigate the TPPE- Hg^2+^ interaction mechanism, DMF-d_7_ was used as a solvent for the proton nuclear magnetic resonance (^1^H NMR) titration during the TPPE-Hg^2+^ complex formation. The coordinative interaction between Hg^2+^ and the pyridyl N atoms in TPPE was detected by the changes in the ^1^H NMR signal. Figure 10 shows the ^1^H NMR spectra of TPPE and of the Hg^2+^/TPPE mixtures at different molar ratios. Upon addition with Hg^2+^, all proton signals were shifted downfield, and the shift of the pyridine ring protons was more pronounced than the one for the phenylene protons. This is an indication of a coordination between the pyridine N donor and Hg^2+^, which will exclude the electron-withdrawing effect by the neighbouring H atoms (i.e., protons). Since the benzene ring is positioned relatively close to the pyridine ring, the proton signals that originate from the benzene ring have also been affected to a certain extent. Furthermore, the H_2_O protons were also significantly shifted during the titration, which implied a fast exchange between the free water molecules and the water molecules that coordinate to Hg^2+^. When the Hg^2+^/TPPE ratio was increased from 1:1 to 2:1, the chemical shifts did not significantly change. This proves that the coordination equilibrium was already established when the metal and ligand were fed at a 1:1 ratio (Fig. S5). This is also consistent with the result from the high-resolution mass spectrometry analysis, which showed a molecular peak corresponding to the [Hg(TPPE)(NO_3_)]^+^ ion (Fig. S6).

### FT-IR

Infrared spectroscopy was used in the study of the interaction between TPPE and Hg^2+^ (Fig. S7). The symmetry and spatial geometry of TPPE was found to change as a result of Hg^2+^ coordination, and a new Hg-N coordination bond was formed. Therefore, the vibrational frequencies of the C–N and C–C bonds that were in close proximity to the Hg-N coordination bond, became altered. The absorption peak of TPPE at 1592 cm^−1^, which is assigned to a ν(C–C)-ν(C–N) composite vibration, shifted to 1595 cm^−1^ as a result of Hg^2+^ coordination. In addition, the main difference was the strong absorption around 1300 cm^−1^, which was attributed to the asymmetric stretching vibrations of the NO_3_^−^ anion^38^.

### Fluorescence and UV–Vis absorption titrations

Additional spectral titration of the TPPE-Hg^2+^ interaction was carried out by using UV absorption and fluorescence spectroscopies and the addition of an increasing amount of Hg ^2+^ to a solution of TPPE (2.0 × 10^−5^ M) in DMF. As shown in Fig. S8b, the fluorescence intensity (λ_em_ = 508 nm) of the solution did not start to rise until the addition of more than one equivalent of Hg^2+^ per TPPE. It reached a stable state after the addition of four equivalents of Hg^2+^, which indicated that the TPPE complex had reached a saturation level. The Job plot in Fig. S8c gives rise to a similar conclusion. The fluorescence intensity increased with the addition of Hg^2+^. However, it did not decrease until a sufficient amount of Hg^2+^ was coordinated, which indicated the presence of a cooperativity effect. This could be attributed to the simultaneous enhancement of coordination and π-π interactions, and the increase in metal ion concentration, which led to an enhanced AIE.

**Figure 11:**
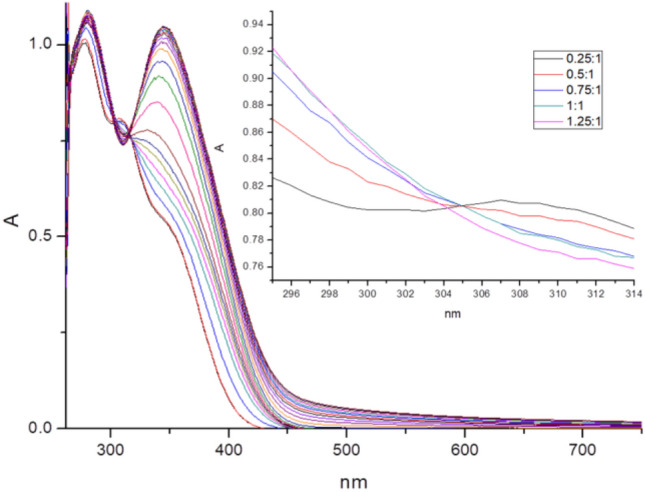
Hg^2+^ UV titration curve. The inlet shows the details in the disappearance of the isoabsorption point for a Hg:TPPE larger than 1:1 (2.0 × 10^−5^ M TPPE in DMF).

The same aggregation phenomenon was revealed by the UV analysis (Fig. S8a, λ = 345 nm). Figure 11 displays the detailed UV response of the TPPE solution towards an increment of Hg^2+^. When going from 1:0 to 1:1, the presence of an isosbestic point at 305 nm became evident. However, this isoabsorption point disappeared upon further addition of Hg^2+^ beyond one equivalent per TPPE. In addition, the absorption peak at 279 nm showed a slight red-shift (to 281 nm) and became broadened. Moreover, the absorbance of the 309 nm band decreased and the band at 339 nm increased in absorption intensity and became finally red-shifted to 345 nm. These results reflect the occurrence of an intermolecular aggregation, as described in the literature^39,40^. The lowest detection limit (LOD) of TPPE for Hg^2+^ was calculated by the 3*δ/S* method^41^, yielding a LOD value of 4.30 × 10^–7^ M (Fig. S9). Also, the association constant was determined as K_a_ = 1.95 × 10^5^ M^−1^, which shows that TPPE is able to detect Hg^2+^ ions with high sensitivity.

### Formation and structural characterization of the metal organogels

**Figure 12:**
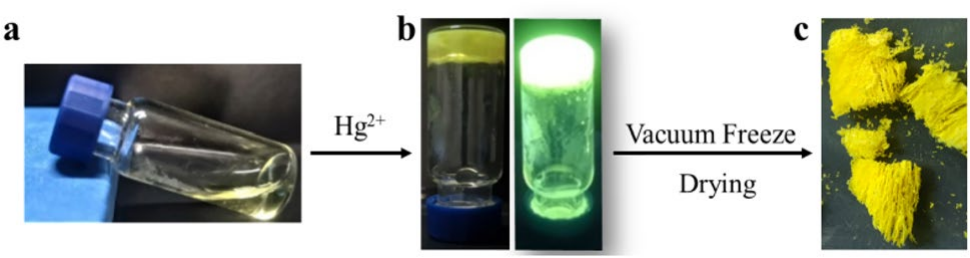
In the DMF solution, a certain concentration of TPPE is added to Hg^2+^ to form a yellow gel (**a**), which becomes bright green under illumination with ultraviolet light (λ_ex_  = 365 nm) (**b**). (**c**) Xerogels exhibit a loose multi-layered structure (TPPE: 9.6 × 10^–3^ M, Hg^2+^: 3.8 × 10^–2^ M).

Interestingly, when the TPPE-Hg^2+^ concentration in the DMF solution was increased (i.e., both TPPE and Hg^2+^), the complex rapidly (within 30 s) formed a yellow metal–organic gel with a very bright fluorescence under UV light in addition to xerogels with a loose multi-layered structure (Fig. 12).

The XRD spectrum of the xerogel is shown in Fig. S10. While it presents a prominent diffusion peak, there are still some diffraction peaks at 2θ = 9.1°, 15.1°, 18.3°, and 27.7°. After a comparison with the standard card of HgNO_3_ (JCPDS: 11–0361), we can assume that these diffraction peaks can be assigned to HgNO_3_. The d value at the maximum of the diffusion peak (2θ = 21.58°) is 4.0478 Å, which indicates that a π-π stacking has occurred within the xerogel.

The TPPE-Hg^2+^ fluorescence was significantly enhanced after the formation of the gel. This may be attributed to the simultaneous enhancement of coordination and π–π interactions with an increase in concentrations, which led to an enhanced AIE (Fig. S11). At the excitation wavelength of 394 nm, the fluorescence intensity of the gel, that is composed of TPPE:Hg^2+^ with a ratio of 1:16 (Int = 3132), became significantly higher than that of the gel obtained with a TPPE:Hg^2+^ ratio of 1:4 (Int = 2518). Also, the emission peak became slightly-blue shifted from 503 to 502 nm at a higher concentration of Hg^2+^. When the excitation wavelength was changed to 439 nm, the fluorescence intensity of the gel composed of TPPE:Hg^2+^  = 1:16 (Int = 4199) also became evidently higher than that of the gel with a TPPE:Hg^2+^ of 1:4 (Int = 2821). Moreover, a blue-shift of the emission peak (from 502 to 498 nm) was again observed for an increased mercury content. The red-shift of the maximum excitation wavelength, when going from the solution to the gel state, has indicated that the excess of mercury ions is involved in the accumulation of aromatic rings. Thus, the increase in mercury ion content may have contributed to the AIE.

### LSCM, SEM, TEM, EDS, ICP, Zeta potential, and BET

**Figure 13:**
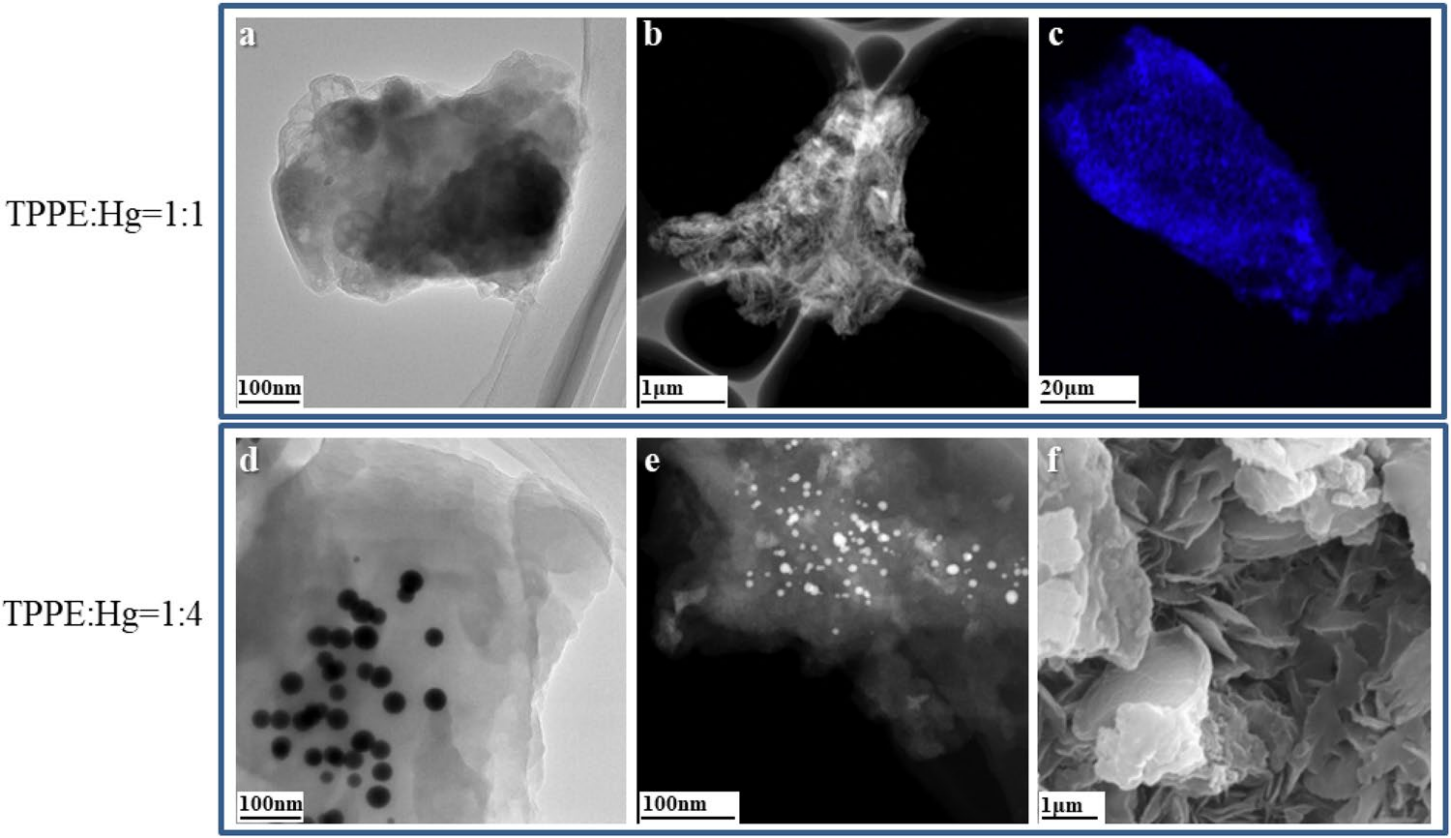
(**a**) Bright field TEM image of the xerogel constructed with a TPPE:Hg of 1:1. (**b**) Dark field TEM image of the xerogel constructed with a TPPE:Hg of 1:1. (**c**) LSCM images of xerogel constructed with TPPE:Hg = 1:1. (**d**) Bright field TEM image of the xerogel constructed with a TPPE:Hg of 1:4. (**e**) Dark field TEM image of the xerogel constructed with a TPPE:Hg of 1:4. (**f**) SEM image of the xerogel constructed with a TPPE:Hg of 1:4.

In order to gain an in-depth understanding of the gel morphology, transmission electron microscopy (TEM) measurements were conducted for the metal–organic gels with a TPPE:Hg ratio of 1:1 and 1:4 (as shown Fig. 13). The images of the gels revealed a stacked multi-layered sheet morphology, which was consistent with the XRD-based assumptions that the complexes will aggregate under the influence of π-π stacking interactions. Laser Scanning Confocal Microscope (LSCM) images showed a bright fluorescence of the gels under UV excitation (Fig. 13c).

**Figure 14:**
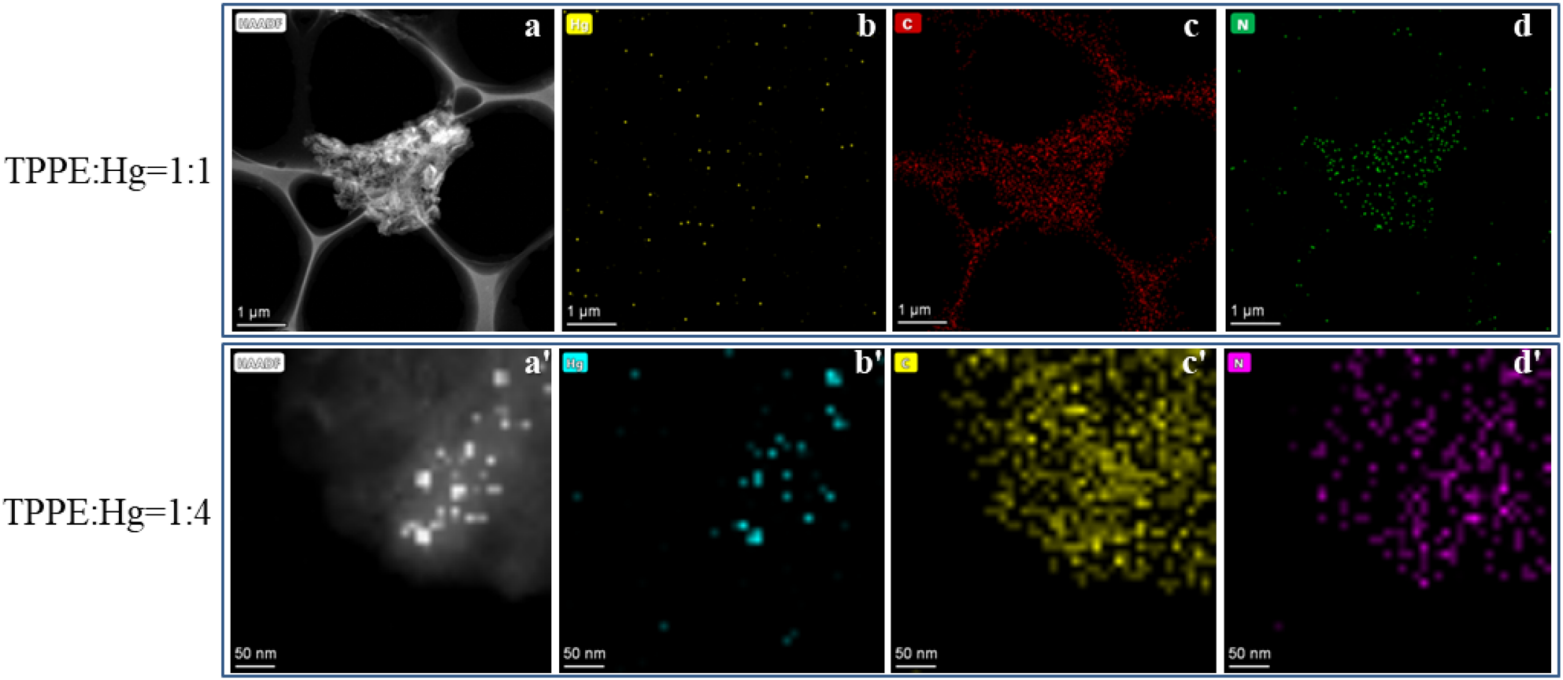
(**a**–**d**) STEM images of the xerogel constructed with a TPPE:Hg of 1:1. (**a'**–**d'**) STEM images of the xerogel created with a TPPE:Hg of 1:4.

For the 1:4 gel, the multi-layer stacked gel adsorbed an excess of Hg^2+^ to form nanocrystals with diameters of 20–30 nm. Bright and dark field images clearly showed that the mercury nanocrystals were uniformly distributed in this layered gel. Scanning TEM (STEM) analyses were carried out on the 1:1/1:4 xerogels, to further prove that mercury nanocrystals were adsorbed in the gel. An exclusive composition of C, N, and Hg elements was then confirmed (Fig. 14). For a TPPE:Hg ratio of 1:1, the elemental mapping analysis did not reveal any obvious mercury content in the aggregated state (Fig. 14b). On the other hand, for the 1:4 material, the high-angle annular dark field SEM (HAADF) image (Fig. 14b') revealed bright spots with a size consistent with those revealed by the TEM analysis (Fig. 13d).

Scanning electron microscopy (SEM) measurements were also carried out to visually obtain the morphological structure of the gel (TPPE:Hg = 1:4). As shown in Fig. 13f, part of the gel surface shows a sheet-like morphology with the sheets arranged in a flower-like shape, and other parts are composed of agglomerated blocks.

The composition of the TPPE-Hg^2+^ coordination polymers (1:1) was further confirmed by using X-ray energy dispersive spectroscopy (EDS). The xerogel X-ray diffraction pattern was found to only reveal a halo (broad “hump”), which was an indication of an amorphous state. This also suggested a random growth when forming the 3-D polymer network during self-assembly. The EDS measured C:N:Hg ratios were 44:5:1 (using TEM, Fig. S12a) and 46:9:1 (using SEM, Fig. S12b), which are in good agreement with the ideal 46:4:1 stoichiometry. It also proved that the TPPE:Hg^2+^ coordination ratio was 1:1 in the coordination polymer.

After the preparation of the organometallic dry gel, according to the 1:4 TPPE:Hg^2+^ ratio, a small amount of the dry gel was placed in water and stirred at room temperature for 24 h. An ICP-OES analysis of the solution showed that about 75% of the Hg^2+^ ions were released, proving that the gel could adsorb an excess of mercury ions (Table S1). In addition, when the 1:4 xerogel was dispersed in a 1:19 mixture of DMF and ethanol, the solution Zeta potential measurement indicated that the gel had a positively charged surface, which suggested a presence of adsorbed metal cations (Table S2). In order to assess the ingestion capacity of the MOG for other heavy metals, the adsorption of Cd^2+^ onto the gel was investigated by the following methods. A portion of the gel and the 500 mg∙L^−1^ Cd^2+^ aqueous solution were mixed in a vial (TPPE:Hg^2+^:Cd^2+^  = 1:2:2). The vial was left to stand for 24 h at room temperature. The ICP-OES analysis verified that the concentration of the residual Cd^2+^ was 78.3 mg∙L^−1^, indicating that the metal organic gel formed by TPPE and Hg^2+^ could further effectively absorb and remove 84.3% of Cd^2+^ (Table S3).

**Figure 15:**
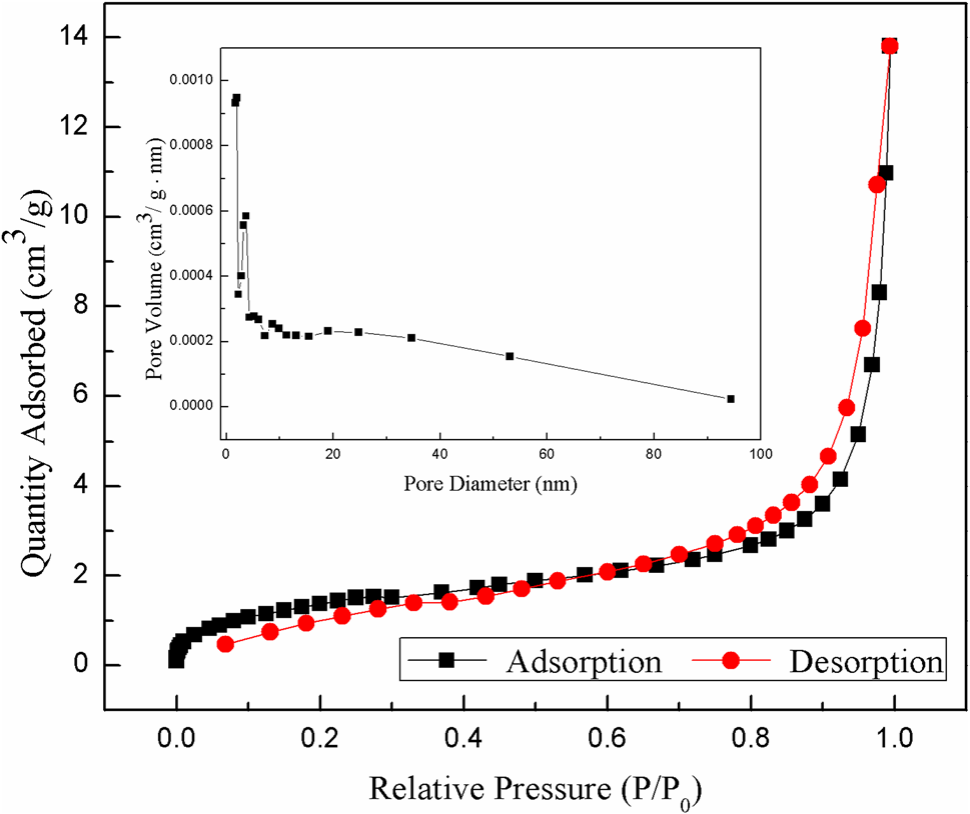
Nitrogen adsorption–desorption isotherms of the MOG at 77 K. The inlet shows the pore size distribution of MOG determined by BJH method.

The porosity of the aerogels was further analyzed by adsorption isotherms of N_2_ measured at 77 K. While the isotherm given in Fig. 15 was examined, it was concluded that the MOG showed a type IV-H3 hysteresis loop according to the International Union of Pure and Applied Chemistry (IUPAC) classifcation. These hysteresis loops are characteristic of layered mesoporous materials^42^, consistent with the results of Barret-Joyner-Halenda (BJH) distribution. We, thereby, assume that after the formation of a metal–organic gel from a 1:1 ratio of TPPE and Hg^2+^, additional heavy metal ions can be further adsorbed in the layered 3-D polymer network. The reason for the formation of these nanocrystal aggregates is not yet clear, and we will continue to investigate it in the future.

### Test kit

**Figure 16:**
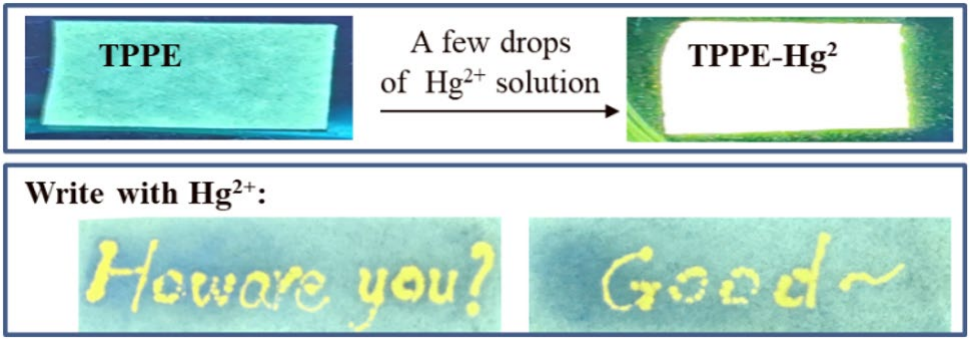
The effect of TPPE as a Hg^2+^ detection kit. Filter paper strips immersed in TPPE solution were dried and used as test strips and detected using a black-box UV analyzer (λ_ex_  = 365 nm).

In order to increase the use of the TPPE probe, test strips were prepared and used to detect Hg^2+^ ions. As shown in Fig. 16, filter paper strips were first immersed in a solution of TPPE in DMF: H_2_O (3:7, v/v) and then dried in air. As the next step, Hg^2+^ was added to the test kit which led to a significant color change that could easily be detected by using a black-box UV analyzer (365 nm). By dipping in the Hg^2+^ solution and writing on the test kit, the writing can be clearly observed under a UV light. Therefore, the filter paper strips can be a convenient test kit for the detection of Hg^2+^ ions.

## Discussion

Because of the aggregation-induced luminescence effect, it was found that TPPE can achieve a highly selective fluorescence of Hg^2+^ in an aqueous solution containing 30% of DMF and results in MOG at high concentrations in DMF. In relation to TPPE, the fluorescence intensity was increasing with an increase in Hg^2+^ from 0 to 4 equivalents. The immobilization of Hg^2+^ is, then, affected by different assembly processes. Since the Hg-N coordinative bond constitutes the strongest interaction, it is reasonable to assume that there is a coordinative building unit that maximizes the Hg-N bonds during the assembling process. For the Hg/TPPE 1:1 system, the assembly of the initially formed molecular TPPE-Hg^2+^ moieties may be attributed to the π–π stacking of the ligand molecules. After the construction of the multi-layer 3-D network structure, the gel can further adsorb heavy metal ions. Although the reason for the formation of mercury nanocrystal aggregates is not yet clear, multiple experimental repetitions yielded similar results, which we will continue to investigate in the future. The application of fluorescent metal–organic gel materials that are based on the AIE principle, has been expanded to heavy metal adsorption.

## Methods

### Materials

All cations (Al^3+^, Ba^2+^, Mn^2+^, Ca^2+^, Fe^3+^, Cu^2+^, Ag^+^, Cd^2+^, Co^2+^, Ni^2+^, Mg^2+^, Pb^2+^, Zn^2+^and Cr^3+^) were purchased in the form of perchlorate salts, except for Hg^2+^ which was purchased in the form of a nitrate. Moreover, all salts were purchased from Alfa-Aesar Chemical (99.0–99.999% metals basis), and stored in a vacuum desiccator before used without further purification. Other reagents and solvents were analytically pure and were used as received without further purification.

### Instruments

The ^1^H NMR spectra were recorded by using a Bruker AVANCE NEO 500 instrument, and the IR measurements were carried out by using a Digilab FTS-3000 FT-IR spectrophotometer. Moreover, UV–vis absorption spectra and fluorescence spectra were recorded by using a Shimadzu UV-2550 and a Shimadzu RF-5301 spectrophotometer, respectively. Also, the pH measurements were carried out in air at room temperature by using a Sartorius PB-10 acidometer. Furthermore, the mass spectra were recorded by using a Bruker Esquire 6000 MS instrument. SEM images were obtained by using a Czech TESCAN MIRA LMS microscope, and TEM images were obtained by using a FEI Talos F200X transmission electron microscope (with an acceleration voltage of 200 kV). Moreover, LSCM images were obtained by using a Zeiss LSM800 instrument, and the ICP-OES data were obtained by using an Agilent 5110 instrument. A Dandong Baite Bettersize2000W zetasizer was used in the determination of zeta potential values. N_2_ adsorption/desorption isotherms was obtained on a Micromeritics ASAP 2460 instrument. The pore size distributions was derived from the adsorption branches of the isotherms by using the Barrett-Joyner-Halenda (BJH) model. Also, a Panalytical Empyrean powder X-ray Cu-Ka radiation diffractometer (λ = 0.15406 nm) was used for structural determinations at a voltage of 45 kV, a current of 40 mA, and a scanning speed of 2 min^−1^ in the 10º ≤ 2*θ* ≤ 70º range. Mention the excitation wavelength for the used UV lamp in the caption of the Figure, wherever it is required.

### Synthesis of TPPE

TPPE was synthesized by using the method presented in Ref.^43^, and it was characterized by using IR and ^1^H NMR. The obtained IR and ^1^H NMR spectra were found to match with those reported in the literature.

### General procedure for the spectroscopic measurements

A solution of TPPE (2.0 × 10^–4^ M) in DMF/H_2_O (3:7, v/v) was prepared and stored in a dry atmosphere. Unless otherwise specified, the variations in the UV–Vis and fluorescence spectra of TPPE were observed upon addition of salts while keeping the TPPE concentration constant (2.5 × 10^–5^ M).

## Supplementary Information

Supplementary Information.

## Data Availability

All data generated or analyzed during this study are included in this published article.
