# Supplementary material for: Hg(II) immobilization and detection using gel formation with tetra-(4-pyridylphenyl)ethylene and an aggregation-induced luminescence effect
Source: Sci Rep. 2023 Feb 6;13:2135. doi: 10.1038/s41598-023-29431-0 (PMC9902491; doi:10.1038/s41598-023-29431-0)
Supplement: Supplementary file 1 — Supplementary Information. [file 41598_2023_29431_MOESM1_ESM.pdf]

Supporting Information

Hg(II) immobilization and detection using gel formation with tetra-(4-pyridylphenyl)ethylene and an aggregation-induced luminescence effect

Bing Hu[1]*, Taibao Wei[2], Yanjun Cui[1], Xia Xu[1], Qiao Li[3]

[1]*College of Science, Gansu Agricultural University, Lanzhou, Gansu, 730070, P. R. China*

[2]*College of Chemistry and Chemical Engineering, Northwest Normal University, Lanzhou, Gansu, 730070, P. R. China;*

[3]*College of Chemical Engineering，Lanzhou University of Arts and Science，Lanzhou, Gansu, 730070, P. R. China*

Figure Captions

Figure S1 Fluorescence and colorimetric identification of different concentrations of Hg^{2+} in DMSO and DMF by TPPE under UV light and visible irradiation.

Figure S2 UV-Vis spectra for different concentrations of TPPE in a) DMF and b) DMSO, before and after the addition of Hg^{2+} ions.

Figure S3 Variations in the fluorescence spectrum of Hg^{2+} (in DMF) with different ethanol contents, as detected by using TPPE.

Figure S4 The effect of Co^{2+} coexistence in DMF/H_2O binary solution on the fluorescence intensity of Hg^{2+} detected by TPPE at different water contents and the photos under UV light.

Figure S5 Changes in chemical shifts during ^1H NMR titration of TPPE with Hg^{2+} using DMF-d_7 as solvent.

Figure S6 HR-ESI-MS spectrum of $[Hg(TPPE)(NO_3)]^+$.

Figure S7 FT-IR spectrum of TPPE and TPPE-Hg.

Figure S8 a) Graph of absorbance at 345nm by UV titration. b) Graph of emission intensity at 508 nm by fluorescence titration. c) Fluorescence JOB curve.

Figure S9 The image of the linear range.

Figure S10 P-XRD spectrum of TPPE and TPPE-Hg xerogel.

Figure S11 Fluorescence intensities of gel state at different metal contents/excitation wavelengths.

Figure S12 a) TEM-EDS and b) SEM-EDS images of xerogel constructed with TPPE:Hg=1:1.

Table S1 ICP-OES analysis of Hg^{2+} release.

Table S2 Zeta potential value.

Table S3 ICP-OES analysis of Cd^{2+} adsorption

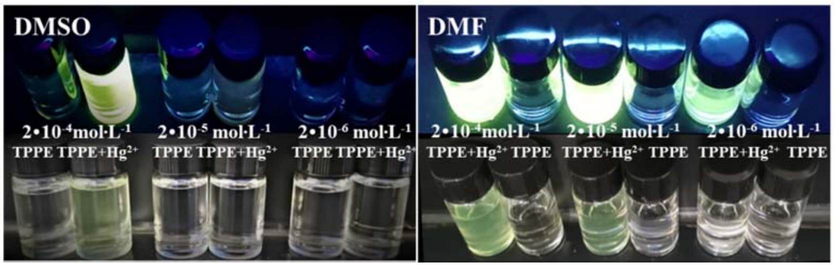

Figure S1 Fluorescence and colorimetric identification of different concentrations of Hg^{2+} in DMSO and DMF by TPPE under UV light and visible irradiation.

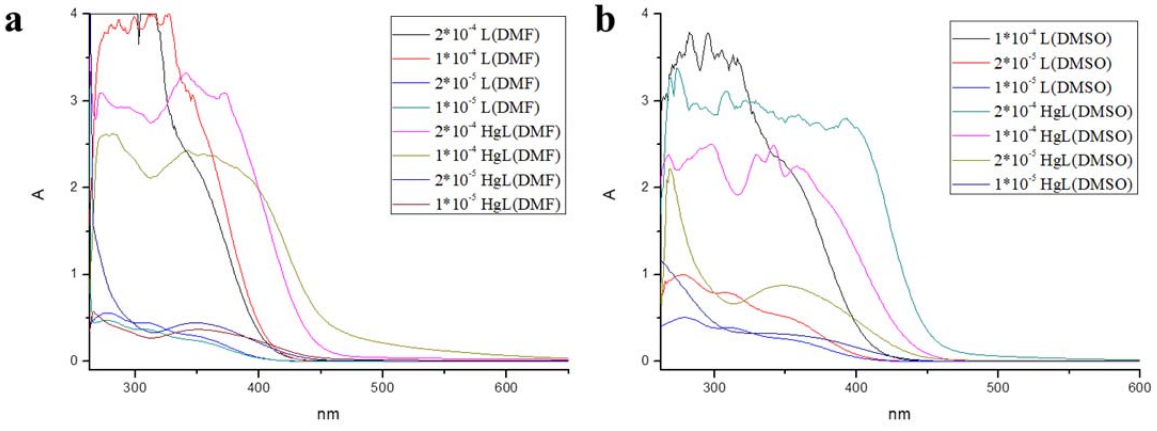

Figure S2 UV-Vis spectra for different concentrations of TPPE in a) DMF and b) DMSO, before and after the addition of Hg^{2+} ions.

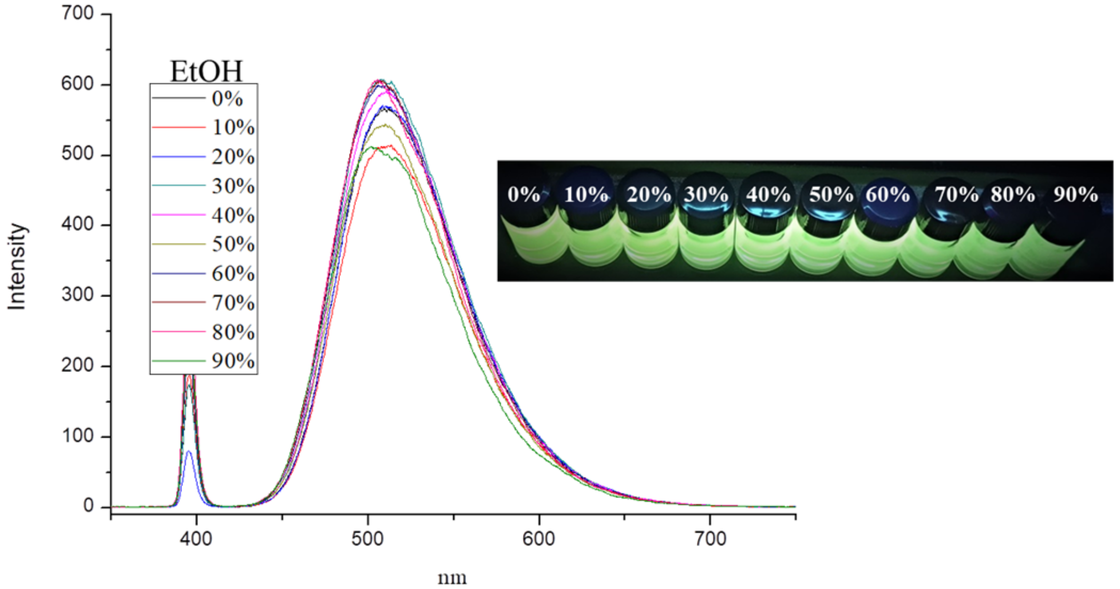

Figure S3 Variations in the fluorescence spectrum of Hg^{2+} (in DMF) with different ethanol contents, as detected by using TPPE (λex = 394 nm) .

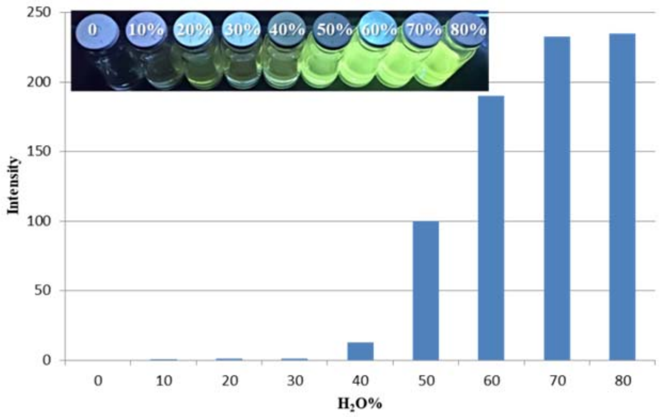

Figure S4 The effect of Co^{2+} coexistence in DMF/H_2O binary solution on the fluorescence intensity of Hg^{2+} detected by TPPE at different water contents and the photos under UV light.

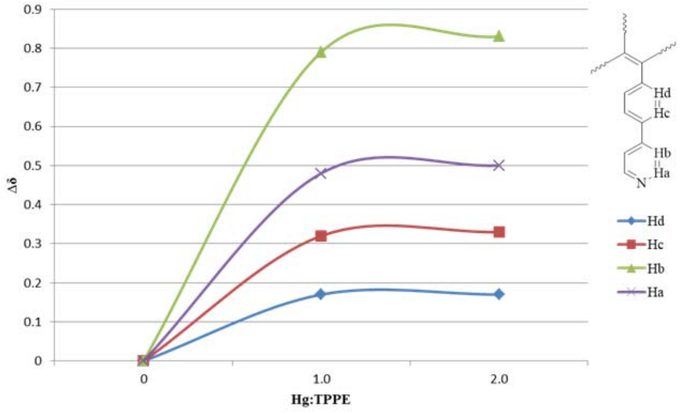

Figure S5 Changes in chemical shifts during ^{1}H NMR titration of TPPE with Hg^{2+} using $DMF\text{-}d_7$ as solvent.

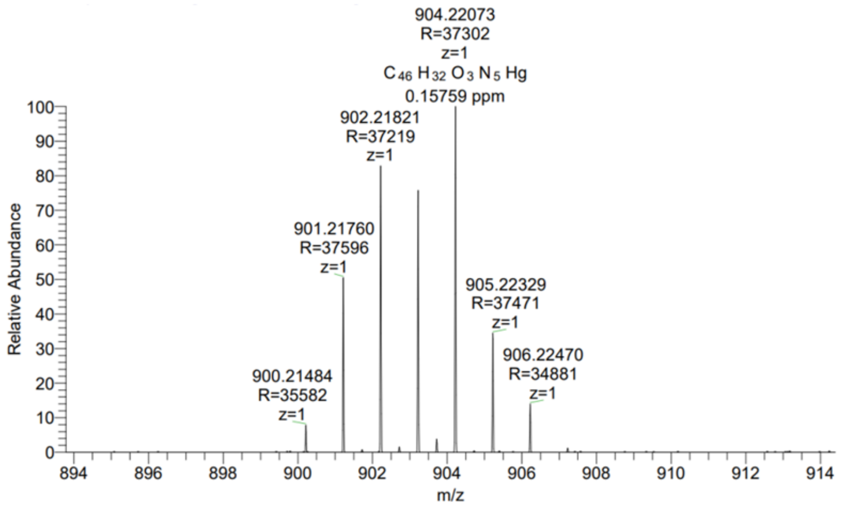

Figure S6 HR-ESI-MS spectrum of $[Hg(TPPE)(NO_3)]^{+}$.

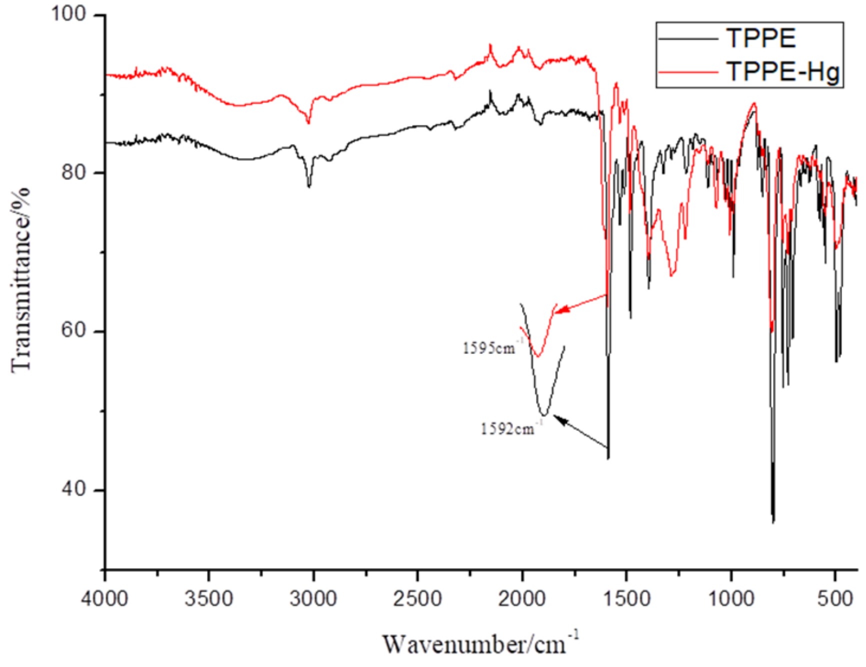

Figure S7 FT-IR spectrum of TPPE and TPPE-Hg.

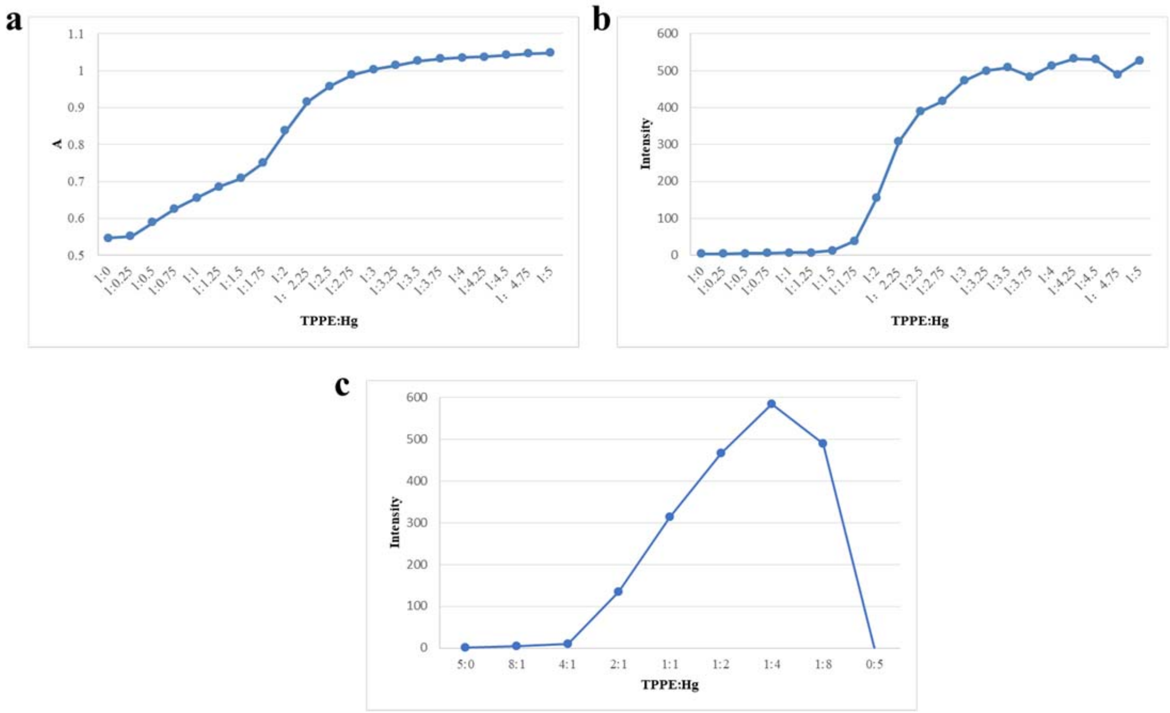

Figure S8 a) Graph of absorbance at 345nm by UV titration.
b) Graph of the emission intensity at 508 nm by fluorescence titration.
c) Fluorescence JOB curve.
($2.0{\times}10^{-5}$ M TPPE in DMF)

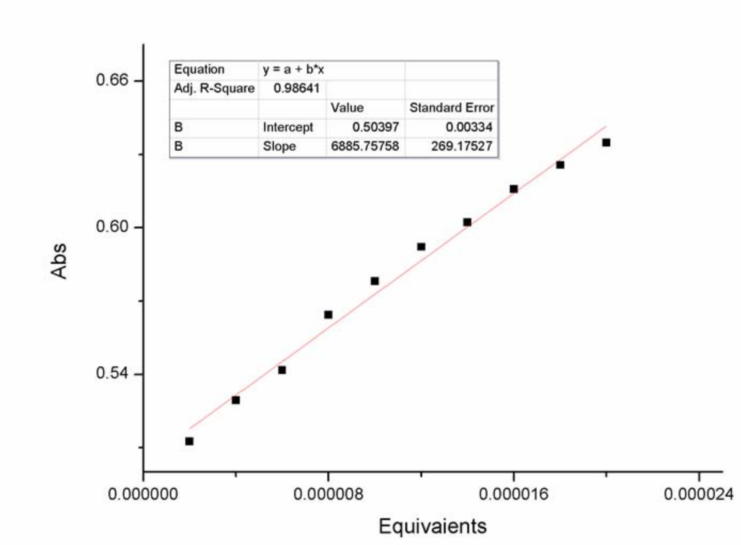

Figure S9 The image of the linear range.
Linear Equation: Y =0.50397 + 6885.75758X
LOD = K × δ/S = 4.30×10^{-7} M （K = 3, δ = 0.000987, S = 6885.7576）

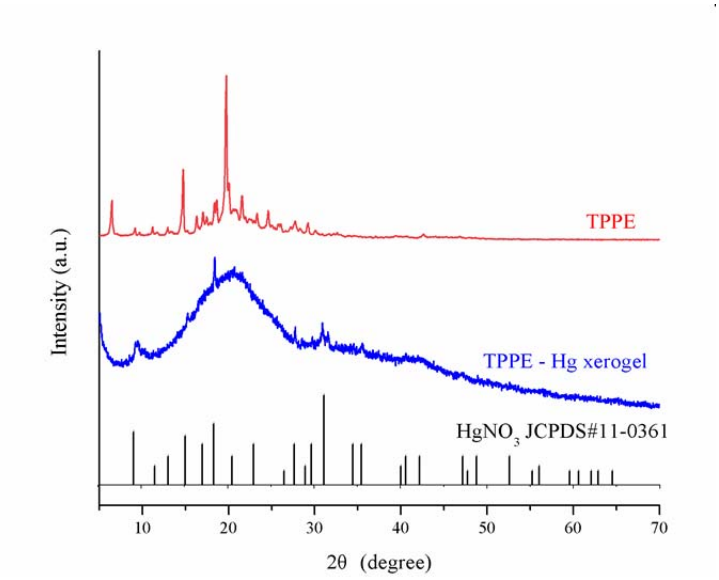

Figure S10 P-XRD spectrum of TPPE and TPPE-Hg xerogel.

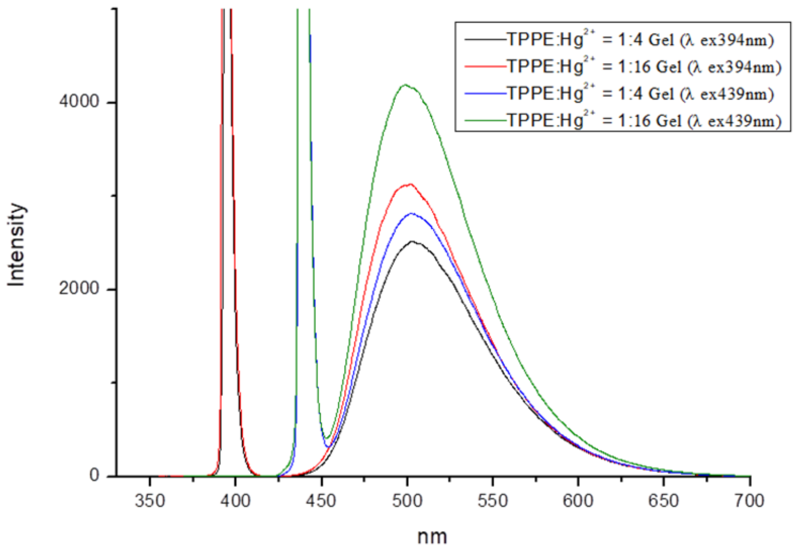

Figure S11 Fluorescence intensities of gel state at different metal contents/excitation wavelengths.
(TPPE: 9.6×10^{-3} M)

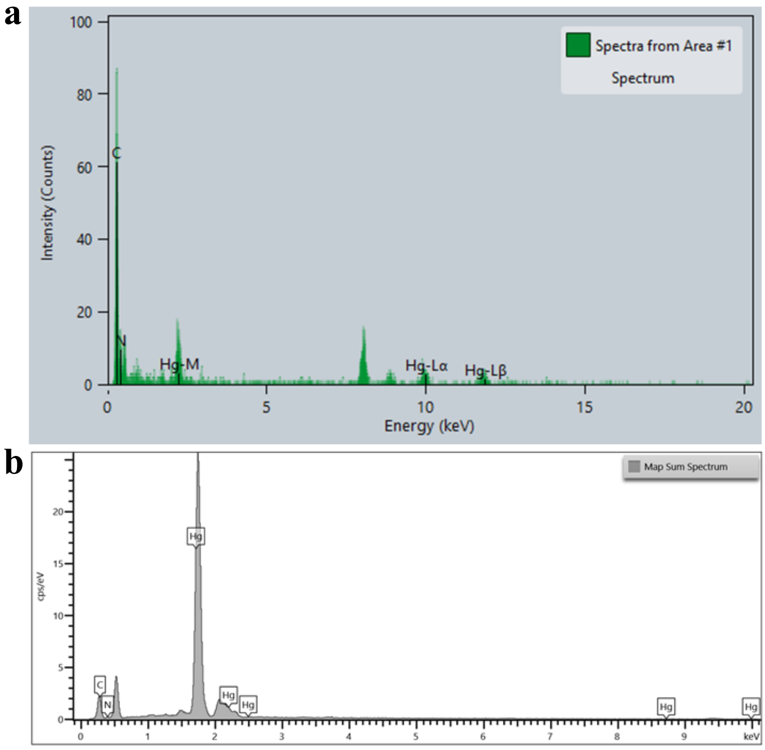

Figure S12 a) TEM-EDS and b) SEM-EDS images of xerogel constructed with TPPE:Hg=1:1.

Table S1 ICP-OES analysis of Hg^{2+} release

Instrument parameters					
		Pump Rate	100r/min	RF Power	1150w
Instrument parameters		Nebulizer Flow	28.0psi	Low WL Range	10s
		Auxiliary Gas	0.5ipm	High WL Range	5s
		Sample Flush Time	20s		
Sample No.	Test element	Element content of sample（mg/L)	Sample element content in 5ml solution（mg)	Theoretical masses of elements in the sample（mg)	
1[a]	Hg	92.7	0.4635	0.68	
2[a]	Hg	92.8	0.4640	0.68	

[a] 0.0032g of TPPE and 0.0068g of mercury salt were used to prepare 0.0064g of metal-organic xerogel. 0.001g xerogel was placed in 5 ml of water, and stirred at room temperature for 24h, then filtered through a 0.45μm membrane and detected the supernatant.

Table S2 Zeta potential value

Testing	1	2	3	average
Zeta potential	13.25	14.14	13.90	13.76

Table S3 ICP-OES analysis of Cd^{2+} adsorption

		Pump Rate	100r/min	RF Power	1150w
Instrument parameters		Nebulizer Flow	0.70L/min	Plasma gas	12.0L/min
		Auxiliary Gas	1.0L/min	Stable Time	20s
		Sample Flush Time	20s	Reading access time	5s
Sample No.	Test element	Initial concentration (mg/L)	Residual concentration（mg/L)	Absorbing rate %	
1	Cd	500	78.3	84.3	
